# Assessing Health-Related Quality of Life of Adult Patients with Intermediate Burns: The Added Value of an Itching and Cognition Item for the EQ-5D: A Retrospective Observational Study

**DOI:** 10.3390/ebj3020023

**Published:** 2022-03-30

**Authors:** J. Nicolaas Dijkshoorn, Juanita A. Haagsma, Cornelis H. van der Vlies, M. Jenda Hop, Margriet E. van Baar, Inge Spronk

**Affiliations:** 1 Burn Centre, Maasstad Hospital, 3079 DZ Rotterdam, The Netherlands; dijkshoornj@maasstadziekenhuis.nl (J.N.D.); vliesc@maasstadziekenhuis.nl (C.H.v.d.V.); 2 Department of Public Health, Erasmus MC, University Medical Centre Rotterdam, 3000 CA Rotterdam, The Netherlands; j.haagsma@erasmusmc.nl (J.A.H.); baarm@maasstadziekenhuis.nl (M.E.v.B.); 3 Trauma Research Unit Department of Surgery, Erasmus MC, University Medical Centre Rotterdam, 3000 CA Rotterdam, The Netherlands; 4 Department of Plastic and Reconstructive Surgery, Erasmus MC, University Medical Centre, 3000 CA Rotterdam, The Netherlands; m.hop@erasmusmc.nl; 5 Association of Dutch Burn Centres, Maasstad Hospital, 3079 DZ Rotterdam, The Netherlands; 6 Dutch Burns Foundation, 1940 EA Beverwijk, The Netherlands

**Keywords:** burns, health-related quality of life, EQ-5D, pruritus, cognition, psychometric analyses

## Abstract

The EQ-5D is increasingly used to assess the health-related quality of life (HRQL) of adult patients with intermediate burns. However, this generic instrument may lack sensitivity, as important problems for burn patients, such as itching and cognition problems are not included in this instrument. This retrospective observational study investigates the value of adding an itching and cognition item to the EQ-5D-3L. Patients completed the EQ-5D-3L, and the Patient and Observer Scar Assessment Scale (POSAS), including an itching item and an extra cognition item three months postburn. The potential added value of an itching and cognition item was studied by distribution, informativity, convergent validity, dimension dependency, and explanatory analyses. In total, 120 patients were included of whom 65% reported itching and 23% reported cognition problems. Adding an itching item to the EQ-5D improved the discriminatory power and informativity of the EQ-5D-3L, but barely increased the explanatory power (0.4%) and decreased the convergent validity (r = −0.529 vs. r = −0.612). In contrast, adding a cognition item slightly improved the informativity and discriminatory power. Moreover, convergent validity (r = −0.617 vs. r = −0.612) and explanatory power increased (4.0%). In conclusion, adding an itching item to the EQ-5D-3L provides some additional information, however, the added value is small, whereas adding a cognition item improved the measurement properties of the EQ-5D-3L in our sample and should be considered when assessing HRQL in adult patients with intermediate burns.

## 1. Introduction

Substantial improvements in burn care have led to a shift in outcomes assessed, from short-term critical care outcomes towards longer-term patient-reported outcomes focusing on mental and physical consequences [1,2,3]. Health-related quality of life (HRQL) is an important and increasingly studied patient-reported outcome to understand, qualify and quantify the impact of burns and assess patient values [4]. HRQL reflects a patient’s perception of how their physical, psychological and social wellbeing is affected by an injury or disease [5].

HRQL is generally assessed with a generic or disease-specific instrument. Burn-specific instruments, such as the Burn Specific Health Scale Brief (BSHS-B), assess specific consequences of burn injuries [6], but outcomes cannot be compared against other populations nor are standard population norms available, impeding the comparison and benchmarking of results with other populations. In contrast, generic instruments, such as the EQ-5D, enable these comparisons but may lack sensitivity for burn-specific problems [7]. Important advantages of the EQ-5D are its short form, so it can be applied worldwide as shown by a recent burn study in Tanzania [8], its availability in many languages, and the ability to assess the burden of disease by calculating DALYs [9] and cost-effectiveness of burn care by calculating QALYs [10]. However, because it does not include burn-specific problems, such as itching, disfigurement, fatigue, or cognition, a (potential) limitation of the EQ-5D is its seemingly limited sensitivity for burn-specific problems [11,12].

To overcome this potential lack of sensitivity and comprehensiveness, an increasing number of studies are investigating the additional value of extra so-called ‘bolt-on’ items to the EQ-5D to cover HRQL in specific populations [13]. An earlier study was performed as proof of concept and showed that this approach is applicable for the burn population [14,15]. A ‘bolt-on’ is a specific item (like itching) about a specific health problem that is not included in the original instrument [16]. Testing the potential added value of a burn-specific bolt-on improves our insights into whether the EQ-5D suffices for patient-reported outcome measurement in burn patients.

A commonly assessed burn-related problem that is not included in the EQ-5D is itching [17,18,19]. A large part of the burn population experience itching, especially shortly after their injury, with prevalence rates up to 87% at three months postburn [19]. Our earlier study showed that the added value of an itching item is small when HRQL is assessed 5–7 years postburn. However, both the prevalence and severity of itching are larger in the acute phase of burns and early in recovery [19]. It is therefore important to assess the potential added value of an itching item to the EQ-5D shortly after sustaining burns.

Within burns, the EQ-5D is sometimes applied with an extra cognition item as it is assumed to increase the sensitivity and comprehensiveness of the EQ-5D for burns [7,20,21]. However, this has not yet been formally studied [11]. Previous studies showed that cognition problems are negatively associated with burn severity [20,22]. Furthermore, a recent study showed that 35% of the burn patients did not recover to their pre-burn level of cognition at 18 months postburn [21]. The proportion of patients not reaching their pre-burn level was highest for the items of cognition and anxiety/depression compared to the other EQ-5D items (4–31%). Cognition problems thus play a pivotal role in the recovery of HRQL of burns patients. In the general trauma population, the added value of extending the EQ-5D with a cognition item has been studied and was shown to improve the psychometric performance of the EQ-5D [13,23]. It is thus important to assess the potential added value of a cognition item to the EQ-5D. Therefore, the aim of this study was to assess to what extent an itching and cognition item add value to the EQ-5D for HRQL assessment in adult patients with intermediate burns three months after their injury.

## 2. Methods

### 2.1. Study Design, Setting and Participants

The current study is a retrospective observational study. Between December 2011 and March 2013, a multicenter RCT study primarily aimed at studying the cost-effectiveness of Laser Doppler Imaging (LDI) was conducted in the three Dutch burn centers (Red Cross Hospital Beverwijk, Martini Hospital Groningen, and Maasstad Hospital Rotterdam) [24]. Patients in the LDI-study were randomly assigned to (1) clinical assessment plus LDI or (2) clinical assessment alone. An LDI scan was obtained of all intermediate-depth wounds by a trained research physician/nurse, between 48 h and 5 days postburn. The LDI results of the first patient group were handed to the treating clinician, where LDI results of the second patient group remained blinded. Additional information regarding Dutch burn care and the population treated is described elsewhere [25]. The study was conducted according to the principles of the Declaration of Helsinki, approved by the Ethics Committee of Rotterdam (NL37844.101.11; 28/10/2011) and the institutional review board of the three participating hospitals, and registered at ClinicalTrials.gov (NCT01489540). This study was reported according to the ‘Strengthening the Reporting of Observational studies in Epidemiology’ (STROBE) guidelines.

In Dutch burn care, LDI scanning is used to inform treatment strategies of burn wounds with unknown depths. Therefore, patients with intermediate-depth burns (the burn wound is not obviously full-thickness or superficial), admission or outpatient treatment, and presentation within five days postburn were included in the present study. Exclusion criteria were the presence of full-thickness wounds next to intermediate wounds, topical treatment/dressings impairing scanning, patients with peri-orbital facial burns, no informed consent, and/or a total body surface area (%TBSA) > 20% [10,24].

To limit patient burden, the present study uses the data from this study as secondary data to assess to what extent an itching and cognition item adds value to the EQ-5D for HRQL assessment in adult burn patients three months after their injury. The inclusion criteria for the present study included adult patients (≥18 years old) with a follow-up measurement at three months postburn. We included only adult patients as parents of children with burns completed a different HRQL instrument [24].

### 2.2. Measures

At three months post-burn, patients had a standard wound consultation at the outpatient clinic and completed a survey that included the EQ-5D-3L. This instrument included the five standard dimensions of the EQ-5D (mobility, self-care, usual activities, pain/discomfort, and anxiety/depression) [16,26]. All dimensions were scored on a 3-point scale: no problems, moderate problems, and extreme problems/unable to [27]. Based on the five standard dimensions, a health profile was created by assigning an ordinal number to each level for each dimension, e.g., 1,2132. Furthermore, a level sum score ranging 5–15, and a summary score based on the Dutch value set ranging from 0 (health status comparable to death) to 1 (perfect health) were calculated [28]. The participants also reported their health on a visual analog scale (EQ VAS) with a score ranging from 0 (worst imaginable health) to 100 (best imaginable health) [27].

In this study, an extra item on cognition was added to the EQ-5D questionnaire. This item was described as problems with concentration, memory or IQ [16]. The cognition item was asked like the other dimensions and included the same response options: no problems, moderate problems, and extreme problems.

The survey also included the patient scale of the Patient and Observer Scar Assessment Scale (POSAS) 2.0, which consists of six items: pain, itching, color, thickness, relief and pliability [29,30]. We used the itching item of the POSAS in this study. Patients reported itching on a 10-point scale ranging from 1 (no itch) to 10 (extreme itch). The itching item was transformed into an experimental 3L-bolt-on: 1 = 1 (no itching); 2–6 = 2; 7–10 = 3, assuming 1 to represent no itching and 7–10 to represent extreme itching.

To minimize bias, survey answers were reported by patients and entered in the database by one researcher (MH) and checked by a research nurse. Two other researchers (ND and IS) checked data on abnormalities and missing data before conducting analyses. Patient and clinical characteristics were extracted from the electronic patient file. Patient characteristics included age and sex; clinical characteristics included the location of the burn, aetiology, percentage total body surface area (%TBSA) burned, length of hospital stay and number of surgeries, reconstructive surgeries, wound healing time, scar therapy.

### 2.3. Data Analyses

The sample size calculation was conducted for the primary outcome: wound healing time and resulted in a calculated sample size of 95 per group. With a 5% dropout rate, a total sample size of 200 was required [24]. For the present retrospective observational study on the added value of an itching and cognition item for the EQ-5D, only adult patients were selected. A recent review showed that the added value assessment of the EQ-5D instrument is valid from sample sizes including >60 patients [13]. We included adult burn patients who responded to all items of the EQ-5D-3L, the cognition item, the EQ VAS, and the POSAS itching item at three months postburn. We performed a non-response analysis to study the characteristics of responders and non-responders at this follow-up measurement. Descriptive statistics were used to assess the characteristics and the differences were assessed with the Mann–Whitney U tests for continuous variables and chi-square tests for categorical variables.

The separate POSAS itching item and the extra cognition item were used to study the added value of an itching bolt-on and/or a cognition bolt-on for the EQ-5D-3L in the burn population. When comparing the EQ-5D with the EQ-5D+’bolt-on item’, the latter consists of the EQ-5D plus the bolt-on item, either the recoded POSAS itching item, the cognition item, or the itching + cognition item. Distributional effect analyses included a calculation of the number of unique profiles and the determination of the ceiling effect of the EQ-5D-3L and EQ-5D-3L+ ’Bolt-on item’. The ceiling was determined by comparing the proportion of perfect health profiles (i.e., 11111 for the EQ-5D-3L and 111111 for the EQ-5D-3L+’Bolt-on item’). The higher the proportion of perfect health profiles, the higher the ceiling. The effect of itching and cognition on the EQ VAS was studied by comparing the mean EQ VAS for each of the three levels of itching and cognition.

The informativity of the EQ-5D-3L with and without the bolt-on items was assessed by the Shannon index (H’) and the Shannon Evenness index (J’) [31,32]. These indices provide information on the ability of the EQ-5D-3L+’Bolt-on item’ to assess diversity and to discriminate in a specific population [33]. The higher the value of the Shannon index (H’), the more information is captured by the measurement instrument. If H’ is higher with the bolt-on item added, then more information is captured, while J’ reflects the relative informativity expressing the use of the classifications given their potential. Both H’ and J’ should be studied for a good interpretation of informativity when comparing instruments [34].

The convergent validity, the degree to which two measures that are supposed to be related, are in fact related, was assessed. We assessed the convergent validity of the EQ-5D-3L and the EQ-5D-3L+’Bolt-on item’ was compared by calculating the Spearman’s rank correlation coefficient of the EQ VAS with both measures (using the level sum score). Cohen’s criteria were applied to evaluate the strength of association: correlations were strong if r ≥ 0.50, moderate if r ≥ 0.30–0.49, and weak if r ≥ 0.10–0.29 [35]. Mutual relations among all domains were studied to uncover redundancy and dependency patterns. Dominance relations were studied in cross tables by studying profiles with an extreme problem in one dimension (L3: extreme problem) and no problem (L1: no problem) in another dimension [36]. The number and percentage of L1/L3 versus L3/L1 contrasts were studied for all dimensions, including the bolt-on items.

The explanatory power of the bolt-on domain was studied using regression analyses in which the EQ VAS score was used as an external reference. The EQ VAS score was predicted from the EQ-5D-3L dimensions with or without the extra bolt-on item. Univariate analyses were used to test the relation between the dimensions of the EQ-5D-3L+’Bolt-on item’ and the EQ VAS. The levels ‘moderate problems’ and ‘extreme problems’ were used for the prediction of the EQ VAS; the level ‘no problems’ was used as a reference category. Multivariate analyses (using backward selection) were applied with all the EQ-5D-3L domains; with and without the extra bolt-on item(s) included in the model. All analyses were performed for each bolt-on domain. The significance level was set at *p* < 0.05 and IBM SPSS Statistics 25 was used for all analyses.

## 3. Results

### 3.1. Characteristics of Study Population

**Figure 1:**
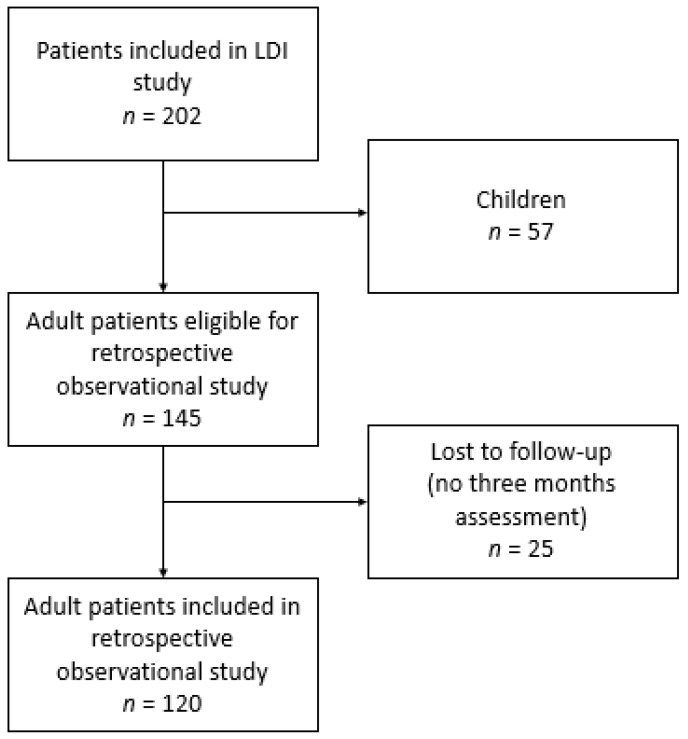
Flowchart on patient selection.

A total of 145 adult patients participated in the original study, 120 (83%) of them completed the survey at three months follow-up and were included in this study (Figure 1). Responders were statistically significantly older (*p* = 0.034) and had a longer hospital stay (*p* = 0.020) than non-responders (Appendix A). The characteristics of the study population are presented in Table 1. Most participants were males (68%), and the median age was 45.0 years old. Patients had a median %TBSA burned of 5.0% and a median length of hospital stay of 12 days. The median wound healing time was 18 days. Slightly less than half of the participants (48%) had to undergo surgery for their burns, and the majority of the burns were caused by flames (56%). The body location burned most often included arms (37%) and hands (28%). The majority of patients (52%) had compression garments, 18% received silicone therapy, 4% had splinting, and two patients had had reconstructive surgery at three months postburn.

### 3.2. Patient-Reported Outcomes

The mean EQ-5D-3L utility was 0.82 (SD 0.21) and the mean EQ VAS was 77.8 (SD 16.5) (Table 2). Patients reported most problems on the pain/discomfort dimension (41%) and least on the self-care dimension (13%). More than half of the patients (65%) reported experiencing slight to extreme itching, and 23% reported experiencing moderate to severe cognition problems.

### 3.3. Distributional Effects

A perfect health status was reported by 47 of the 120 participants (39%) based on the EQ-5D-3L, whereas 24 (20%) participants reported a perfect health status based on the EQ-5D-3L+Itching; 46 (38%) based on the EQ-5D-3L+Cognition; and 23 (19%) based on the EQ-5D-3L+Itching+Cognition. The ceiling was thus highest for the EQ-5D-3L and lowest for the EQ-5D-3L+Itching+Cognition. The 23 participants that had a perfect health status based on the EQ-5D-3L, but not based on the EQ-5D-3L+Itching+Cognition were mainly males (74%). These patients had a median age of 38 years, a median %TBSA of 3.6%, and a median length of hospital stay of 5 days. Their mean EQ VAS was 87.4. 

The mean EQ VAS score for each level of itching and cognition is presented in Table 3. The EQ VAS score clearly decreases with increasing cognition problems, whereas the EQ VAS score was comparable for moderate and severe itching. It is primarily cognition problems, and to a much lesser extent itching, that negatively influence the overall experienced health status of patients.

### 3.4. Informativity of the EQ-5D-3L with and without the Bolt-On Items

In total, 30 out of 243 (12.3%) different health profiles were identified for the EQ-5D-3L, 43 out of 729 (5.9%) for the EQ-5D-3L+Itching, 41 out of 729 (5.6%) for the EQ-5D-3L+Cognition, and 53 out of 2187 (2.4%) for the EQ-5D-3L+Itching+Cognition.

To compare these outcomes and study the informativity of the EQ-5D-3L and the bolt-on items, we calculated the Shannon Index (H’) and the Shannon Evenness index (J’) which take into account the sample size and possible health profiles. The Shannon Index (H’) was 3.62 for the EQ-5D-3L; 4.58 for the EQ-5D-3L+Itching; 3.96 EQ-5D-3L+Cognition; and 4.43 for the EQ-5D-3L+Itching+Cognition. Most information is thus captured by adding the itching bolt-on, the least information was captured by the original EQ-5D-3L instrument.

The Shannon Evenness index (J’) was highest for the EQ-5D-3L+Itching (0.48), followed by the EQ-5D-3L (0.46), followed by the EQ-5D-3L+Cognition (0.42). The Shannon Evenness index (J’) was lowest for the EQ-5D-3L+Itching+Cognition (0.40). These results imply that the informativity increases most by adding an itching item. Most information is thus captured by the EQ-5D-3L+Itching; the EQ-5D-3L+Itching was best to discriminate between different patients in our study population.

### 3.5. Convergent Validity

The Spearman’s rank correlation coefficient was −0.612 (*p* < 0.001) between the EQ-5D-3L and the EQ VAS; -0.529 (*p* < 0.001) between the EQ-5D-3L+Itching and the EQ VAS; −0.617 (*p* < 0.001) between the EQ-5D-3L+Cognition and the EQ VAS; and −0.548 (*p* < 0.001) between the EQ-5D-3L+Itching+Cognition and the EQ VAS (Table 4). This shows that the convergent validity was highest for the EQ-5D-3L+Cognition, meaning that the EQ-5D-3L with the extra cognition is stronger related to the EQ VAS compared to the EQ-5D-3L. On the other hand, the convergent validity was lowest for the EQ-5D-3L+Itching, showing that the EQ-5D-3L without an extra itching item is stronger related to the EQ VAS.

### 3.6. Dimension Dependency

Combinations of extreme values of the EQ-5D-3L+’bolt-on items’ are presented in Table 5 in absolute numbers and in the percentage of total profiles. No problems on itching and extreme problems on any of the five standard EQ-5D dimensions were uncommon (0–0.8%), whereas extreme itching problems and no problems on other dimensions were relatively common (6.7–15.0%) indicating that the standard dimensions are dominant over itching. For cognition, no clear dominancy pattern was seen as both combinations were relatively uncommon (0–2.5%).

### 3.7. Explanatory Power of the EQ-5D-3L with and without Bolt-On Items

Appendix B shows the univariate regression analyses between the EQ-5D-3L+’bolt-on items’ and the EQ VAS. Both levels of itching were not associated with the EQ VAS, whereas both levels of cognition were associated with the EQ VAS. Table 6 presents the multivariate regression analyses. The standard five dimensions explained 52.9% of the variance of the EQ VAS. The addition of the itching item added only 0.4% of explained variance, whereas the addition of the cognition item increased the explained variance by 4.0%. Adding both the itching and cognition item explained 57.2% of the EQ VAS, and thus, explained 4.3% more than the standard five dimensions. The anxiety/depression dimension of the EQ-5D-3L seems to explain the largest part of the EQ VAS as in both scenarios the explained variance was lowest without the anxiety/depression dimension (42.9% and 52.4%). In contrast, the pain/discomfort dimensions seemed to explain the smallest part as the explained variance was highest without this dimension (52.6% and 56.0%).

## 4. Discussion

The present study investigated to what extent adding an itching and cognition item added value to the EQ-5D for HRQL assessment in adult patients with intermediate burns three months after their injury. Our results demonstrated that adding an itching item does improve the informativity and discriminatory power of the EQ-5D, however, the added value is rather small. The additional itching item improves the ability to discriminate between burn patients, but, in general, itching does not seem to impact patients’ experienced health status (EQ VAS) at three months postburn. Adding a cognition item, on the contrary, slightly improves the informativity and discriminatory power (less than the itching item), but adds more value to the EQ-5D-3L compared to the itching item. The convergent validity was highest between the EQ VAS and EQ-5D-3L+C, and the cognition item adds explanatory power to the EQ-5D. Despite the fact that a smaller proportion of the studied patients experienced cognition problems (23%) compared to itching (65%), especially cognition problems and to a much lesser extent itching influences the overall experienced health status (EQ VAS) of burn patients, and thus, adds value to the assessment of HRQL by the EQ-5D. Adding the itching item next to the cognition item barely improved the explanatory power.

Even though earlier studies reported that itching is prevalent after burns and is sometimes experienced as unbearable [17,18,19,37], the addition of an itching item to the EQ-5D only marginally improved the added value of the EQ-5D in our sample. The itching item did discriminate between patients, but itching did not seem to bother patients that much that it impacts their experienced health status as assessed by the EQ VAS. Moreover, earlier studies showed that some additional information was provided by the extra itching item, but the added value was small in the assessment of HRQL 5–7 years postburn [15], and itching had only a limited effect on sleep and daily activity [38]. However, another study suggested that itching is associated with a lower HRQL of burn patients [37], which might be partly explained by the fact that itching was captured by the pain/discomfort item of the EQ-5D as shown by a previous study [14]. Itching might be bearable and may not bother patients too much due to the fact that many patients received skin rehabilitation and scar prevention treatment at three months postburn; the timepoint at which our study was performed. Earlier studies showed that these scar management strategies decrease itching as well as hypertrophic scarring [39].

Based on the current and previous studies, there seems to be no strong indication to include an itching item for the assessment of HRQL of burn patients. However, the present study only included adult patients with intermediate burns and a TBSA of ≤20%. Earlier studies suggested that the prevalence and severity of itching are associated with the severity of burns; patients who needed surgery for their burns had an increased risk of long-term and persistent itching [19,38]. It is therefore important to study whether an itching bolt-on item adds value in the HRQL assessment of patients with deep and severe burns.

Adding a cognition item to the EQ-5D has been studied in the general trauma population before, however, not specifically in burn patients. Earlier studies investigated the convergent validity of the EQ-5D. Golicki et al. [40], as well as Geraerds et al. [41,42], reported correlation coefficients comparable to ours, with a higher convergent validity when the cognition item was added. In our study, the addition of the cognition item to the EQ-5D-5L increased the explanatory power by 4% which is relatively high compared to other studies in general trauma patients as shown by Ophuis et al. (1%) [36] and Geraerds et al. (0.2–2%) [41,42]. Jelsma et al. found that adding a concentration item resulted in an increased explanatory power by 3% [43]. In comparison, the addition of a cognition item for assessment of HRQL seems highly relevant for the adult burn population with intermediate burns.

Adding an itching item to the EQ-5D discriminated better between patients than adding a cognition item in this study. This can partly be explained by the fact that the proportion of patients who reported itching (65%) was higher than the proportion of patients who reported cognition problems (23%). Despite this difference in prevalence between itching and cognition, the cognition item added unique information to the EQ-5D for the assessment of HRQL in adult patients with intermediate burns, whereas the itching item barely did.

In the present study, we analyzed the added value of two important consequences of burns. These results are important to increase insight into the HRQL of burns patients, and based on our results, it is recommendable to add a cognition item to the EQ-5D for assessing HRQL of burn patients, both in clinical practice and research. Our study included a subgroup of all patients, namely those with intermediate burns, covering about 75% of our Dutch burn population. Based on our study and earlier studies [7,20], the extra cognition item would be of interest to assess in all burn patients. However, the extra itching item might only be relevant for patients with severe burns. Furthermore, other consequences of burns are prevalent in burn patients and may not be captured by the dimensions of the EQ-5D, such as skin/scar problems, heat sensitivity, body image, sexuality, fatigue, etc. [44,45]. It is important to uncover what dimensions patients and clinicians would add to the EQ-5D to assess the HRQL of burns patients. Another recommendation is to have a longer follow-up with more measurement moments and more bolt-on items. That makes it possible to determine if and when specific bolt-on items add value to the assessment of HRQL in burn patients. Furthermore, it would be interesting to study the added value of bolt-on items in specific subgroups of burns patients, such as severely burned patients, patients with a facial burn or children with burns.

### Strengths and Limitations

This study was subject to strengths and limitations. The strengths included the use of validated instruments [29,46] and the prevalence and variation in itching and cognition among participants which allowed us to investigate the added value of extending the EQ-5D-3L with a cognition and itching item. Another strength is the low percentage lost to follow-up (17%), As well as the use of secondary data, thereby minimizing the burden to patients is a strength of this study. However, this can also be considered a weakness as data were collected from 2011 to 2013. Limitations include the inclusion of a subgroup of burn patients, namely those with intermediate burns and ≤20%TBSA burned, which is about 75% of all patients treated in Dutch burn centers. Only 10% of our patients have severe burns [25]. Patients with severe burns might experience more severe itching which might impact their HRQL. An extra itching item might thus be relevant to assess HRQL in this specific subpopulation. Future studies should investigate this. Furthermore, only adult burn patients were included in this study, which can be seen as a limitation. However, in children, parents usually assess the HRQL of their children and different assessment instruments are usually applied. Moreover, responders were significantly older and had larger burns compared to non-responders. This might have led to a potential overestimation of the impact on HRQL [47]. As indicated, especially the added value of an itching bolt-on might be larger in patients with more severe burns as severe itching is associated with severe burns. Therefore, future studies should look into this. Another limitation included the use of the EQ-5D-3L version; the newer 5L version has five response categories rather than three and is somewhat more sensitive, especially for mild problems [34,48]. Mapping of the POSAS 2.0 10-level scale to the EQ-5D 3-level scale can also be considered a limitation. This arbitrary mapping may have invoked measurement bias due to an increased ceiling effect, lack of refinement, and overestimation of reported problems [49]. This may have led to fewer problems reported on the 10-level scale compared to when it would have been assessed at a 3-level scale.

## 5. Conclusions

Adding an itching item to the EQ-5D-3L provides some additional information, however, the added value is small. Whereas, adding a cognition item improved the measurement properties of the EQ-5D-3L in adult patients with intermediate burns, and should be considered when assessing HRQL in burn patients.

## Figures and Tables

**Table 1:** Characteristics of study population.

Characteristics	Total Sample (*n* = 120)
Sex: male, *n*(%)	82 (68.3%)
Age at burn (Median, IQR)	45.0 (29.3–59.8)
%TBSA (Median, IQR)	5.0 (2.0–8.5)
Length of hospital stay (Median, IQR)	12.0 (2.0–20.0)
Surgery, *n*(%)	
Yes	57 (47.5%)
No	63 (52.5%)
Wound healing time (Median, IQR)	18.0 (15.0–23.0)
Reconstructive surgery, *n*(%) ^1^	2 (1.7%)
Body location burned, *n*(%) ^2^	
Head/face	2 (1.7%)
Trunk	12 (10.0%)
Arm	44 (36.7%)
Hand	34 (28.3%)
Legs	22 (18.3%)
Feet	6 (5.0%)
Aetiology, *n*(%)	
Flame	67 (55.8%)
Scald	22 (18.3%)
Other	31 (25.8%)
Scar therapy, *n*(%)	
Compression garments	62 (51.7%)
Silicone therapy	21 (17.5%)
Splinting	5 (4.2%)

M, SD = mean, standard deviation; IQR = interquartile range. ^1^ Two missing values; ^2^ patients’ worst scar body location; patients completed the POSAS for this body location.

**Table 2:** EQ-5D-3L and POSAS Patient Scale outcomes.

Patient-Reported Outcomes	Total Sample (*n* = 120)
EQ-5D-3L scores	
Utility score (M, SD)	0.82 (0.21)
EQ VAS (M, SD)	77.8 (16.5)
Mobility (% with problems)	15.0%
Self-care (% with problems)	12.5%
Usual activities (% with problems)	35.8%
Pain/discomfort (% with problems)	40.8%
Anxiety/depression (% with problems)	27.5%
Cognition (% with problems)	22.5%
POSAS Patient Scale scores	
Itching (Median, IQR)	3.0 (1.0–5.0)
% patients with itching (POSAS itching score ≥ 2)	65.0%

M, SD = mean, standard deviation; IQR = interquartile range

**Table 3:** EQ VAS score for each of the three itching and cognition levels.

Domain Score	Number of Patients	Mean EQ VAS (95% CI)
Itching = 1	42	82.1 (78.6–85.5)
Itching = 2	57	75.2 (70.1–80.3)
Itching = 3	21	76.2 (68.8–83.6)
Cognition = 1	93	81.7 (79.0–84.5)
Cognition = 2	24	66.7 (59.0–74.4)
Cognition = 3	3	43.3 (29.0–57.7)

**Table 4:** Spearman’s rank correlation between EQ VAS and the EQ-5D-3L with and without an extra itching and/or cognition item added.

	EQ VAS	*p*-Value
EQ-5D-3L	−0.612	*p* < 0.001
EQ-5D-3L+Itching	−0.529	*p* < 0.001
EQ-5D-3L+Cognition	−0.617	*p* < 0.001
EQ-5D-3L+Itching+Cognition	−0.548	*p* < 0.001

**Table 5:** Combinations of extreme values of the EQ-5D-3L, itching item and cognition item (no vs. extreme problems) (*n* = 120). Data shown as absolute numbers and expressed as the percentage of the total EQ-5D-3L+Itching or EQ-5D-3L+Cognition profiles.

		No Problems						
		Mobility	Self-Care	Usual Activities	Pain/ Discomfort	Anxiety/ Depression	Itching	Cognition
**Extreme problems**	Mobility		1 (0.8 %)	1 (0.8%)	1 (0.8%)	2 (1.7%)	0	1 (0.8 %)
	Self-care	0		0	0	1 (0.8 %)	0	0
	Usual activities	7 (5.8%)	4 (3.3%)		1 (0.8%)	4 (3.3%)	1 (0.8%)	3 (2.5%)
	Pain/discomfort	2 (1.7%)	2 (1.7%)	1 (0.8%)		1 (0.8%)	0	1 (0.8 %)
	Anxiety/depression	1 (0.8%)	1 (0.8%)	1 (0.8%)	0		0	0
	Itching	18 (15.0%)	16 (13.3%)	9 (7.5%)	8 (6.7%)	12 (10.0%)		17 (14.2%)
	Cognition	3 (2.5%)	3 (2.5%)	1 (0.8 %)	1 (0.8 %)	3 (2.5%)	1 (0.8%)	

**Table 6:** Explanatory power of multivariate models for the EQ VAS that include EQ-5D-3L dimensions with and without an extra itching and/or cognition item added.

Selection of EQ-5D-3L+Itching Items	R^2^	F-Value	*p*-Value
MO+SC+UA+PD+AD	0.529	12.2	<0.001
SC+UA+PD+AD+IT	0.517	11.7	<0.001
MO+UA+PD+AD+IT	0.492	10.5	<0.001
MO+SC+PD+AD+IT	0.481	10.1	<0.001
MO+SC+UA+AD+IT	0.526	12.1	<0.001
MO+SC+UA+PD+IT	0.429	8.2	<0.001
MO+SC+UA+PD+AD+IT	0.533	10.2	<0.001
SC+UA+PD+AD+CO	0.545	13.1	<0.001
MO+UA+PD+AD+CO	0.525	12.1	<0.001
MO+SC+PD+AD+CO	0.536	12.6	<0.001
MO+SC+UA+AD+CO	0.560	13.9	<0.001
MO+SC+UA+PD+CO	0.524	12.0	<0.001
MO+SC+UA+PD+AD+CO	0.569	11.8	<0.001
MO+SC+UA+PD+AD+IT+CO	0.572	10.0	<0.001

Note. MO = mobility, SC = self-care, UA = Usual activities, PD = Pain/discomfort, AD = Anxiety/depression, IT = itching, CO = cognition.

## Data Availability

The dataset used and analysed during the current study are available from the corresponding author on reasonable request.

## Appendix A

**Table A1:** Characteristics of responders versus non-responders.

	Responders (*n* = 120)	Non-Responders (*n* = 25)	*p*-Value
Sex: male, *n*(%)	82 (68.3%)	17 (68.0%)	0.974
Age at burn (Median, IQR)	45.0 (29.3–59.8)	34.0 (27.0–44.5)	0.034
%TBSA (Median, IQR)	5.0 (2.0–8.5)	3.0 (1.0–6.8)	0.084
Length of hospital stay (Median, IQR)	12.0 (2.0–20.0)	3.0 (0.0–9.0)	0.020
Surgery, *n*(%)			0.074
Yes	57 (47.5%)	7 (28.0%)	
No	63 (52.5%)	18 (72.0%)	
Aetiology, *n*(%)			0.808
Flame	67 (55.8%)	13 (52.0%)	
Scald	22 (18.3%)	6 (24.0%)	
Other	31 (25.8%)	6 (24.0%)	

IQR = interquartile range.

## Appendix B

**Table A2:** Univariate analyses of the EQ VAS.

	R-Square	Unstandardized B (95% CI)	*p*-Value
Mobility level 2	0.099	−15.2 (−23.6 to −6.8)	**<0.001**
Mobility level 3	0.067	−33.3 (−56.0 to −10.7)	**0.004**
Self-care level 2	0.089	−15.3 (−24.3 to −6.4)	**0.001**
Self-care level 3	0.142	−68.3 (−98.9 to −37.7)	**<0.001**
Usual Activities level 2	0.086	−10.7 (−17.1 to −4.3)	**0.001**
Usual Activities level 3	0.147	−24.0 (−34.5 to −13.5)	**<0.001**
Pain/discomfort level 2	0.133	−12.3 (−18.1 to −6.6)	**<0.001**
Pain/discomfort level 3	0.020	−14.8 (−33.9 to 4.2)	0.126
Anxiety/depression level 2	0.112	−12.6 (−19.1 to −6.1)	**<0.001**
Anxiety/depression level 3	0.089	−38.4 (−60.8 to −16.0)	**0.001**
POSAS Itching level 2	0.022	−4.9 (−10.9 to 1.0)	0.105
POSAS Itching level 3	0.002	−1.9 (−9.8 to 6.0)	0.633
Cognition level 2	0.113	−13.9 (−20.9 to −6.8)	**<0.001**
Cognition level 3	0.112	−35.3 (−53.4 to −17.2)	**<0.001**
